# An ice-binding protein from an Arctic population of American dunegrass, *Leymus mollis*

**DOI:** 10.12688/f1000research.24328.2

**Published:** 2020-08-17

**Authors:** Todd L. Sformo, James A. Raymond

**Affiliations:** 1 Department of Wildlife Management, North Slope Borough, Utqiaġvik, Alaska, 99723, USA; 2 Institute of Arctic Biology, University of Alaska Fairbanks, Fairbanks, Alaska, 99775, USA; 3 School of Life Sciences, University of Nevada Las Vegas, Las Vegas, Nevada, 89154, USA

**Keywords:** Leymus mollis, ice-binding protein, Arctic, Alaska, Pooidae, dune grass, Chukchi Sea

## Abstract

Several cold-hardy grasses have been shown to have ice-binding proteins (IBPs) that protect against freeze-thaw injury. Here, we looked for IBP activity in an Alaskan coastal grass, *Leymus mollis* (Pooidae), that had not previously been examined. Rhizome tissue had strong ice-structuring and ice recrystallization inhibiting (IRI) activities, indicating the probable presence of IBPs. The gene sequence of an IBP was obtained. The sequence encoded a 118-amino acid IRI domain composed of eight repeats and that was 80% identical to the IRI domain of the IBP of perennial ryegrass *Lolium perenne*. The predicted 3D structure of the IRI domain had eight beta-roll coils like those in *L. perenne* IBP

## Introduction

Within the grass family (Poaceae), the subfamily Pooideae includes many cold-adapted grasses including wheat, barley and forage grasses. These grasses have developed a large family of ice-binding proteins (IBPs) that protect the plants from freezing damage ( Sandve *et al*., 2008). The IBPs, which have negligible effect on the freezing point, are characterized by a C-terminal domain consisting of several repeating units that is associated with strong ice recrystallization inhibition (IRI) activity ( Sandve *et al*., 2008). This region is thus called the IRI domain. Ice recrystallization, in which larger ice grains grow at the expense of smaller grains, occurs mostly at warmer sub-zero temperatures and is thought to cause damage to cell walls. Two of the most studied of these IBPs are from the grasses *Lolium perenne* ( Sidebottom *et al*., 2000) and *Deschampsia antarctica* ( John *et al*., 2009). Their amino acid sequences are very similar in both the N-terminal and IRI domains. The 118-a.a. IRI domain of the *L. perenne* IBP has been crystallized and its structure determined by X-ray crystallography ( Middleton *et al*., 2012). The IRI domain has a beta-roll fold with eight similar coils, one side of which is the predicted ice-binding site. The spacing of the coils is very close to the repeat distance along the *a*-axis of ice. Interestingly, heterologous expression of the *Lolium* IRI domain in tomato was also shown to increase chilling (4°C) tolerance ( Balamurugan *et al*., 2018), although the mechanism remains unclear. Here, we describe a related IBP from a pooid grass from Utqiaġvik (Barrow), Alaska, *Leymus mollis*. This site, at 71.3° N, is the highest latitude at which IBPs have been examined in a grass It is characterized by a short growing season from mid-June to late August with an average air temperature of 4°C ( Tieszen, 1972), and frequent subzero air temperatures (Table S1, *Extended data*).

*L. mollis*, also known as American dunegrass, is found in coastal habitats, especially sand dunes across North America and Greenland. It is typically subjected to many stresses, such as low nutrient levels, salt spray, little freshwater, inundation during storms, wind abrasion, and ice storms ( Gagné & Houle, 2002). Here we characterize the IBP of *L. mollis* and compare it with the IBP of *L. perenne*, a plant that is not as well adapted to such northern regions ( Helgadóttir *et al.,* 2018).

## Methods

### Grass

A grass sample was collected from a gravel beach at Utqiaġvik (Barrow), Alaska (71.3°N) on 6 October 2019, and stored at -80°C. The grass was shipped frozen to University of Nevada Las Vegas for analysis, but accidentally rose to ~10°C for about one day during shipment.

### Activity measurements

To obtain a sample for measuring ice structuring and IRI activities, the brown outer layers of a rhizome from just below the soil surface were peeled off, revealing green tissue underneath. About 70 mg of the green tissue was ground in 1 ml water in a mortar and pestle. The suspension was centrifuged at 14,000 rpm for 5 minutes to yield a slightly cloudy supernatant. Stem tissue from lawn grass ( *Festuca* sp.) at the University of Nevada Las Vegas (52 mg) was similarly homogenized and used as a control. Ice-structuring activity was observed by examining the growth of an ice seed crystal with well-defined ice *c*- and *a*-axes submerged in the grass extract supernatant, as described previously ( Raymond & Fritsen, 2001). Briefly, the sample was placed in a rectangular tube and the tube was submerged in a controlled temperature bath with front and rear windows. The growth of the crystal was observed at a temperature slightly below the freezing point (~-0.2°C) with a horizontally mounted dissecting microscope. Ice-structuring activity was defined as the appearance of sharply defined facets on the crystal surface. IRI activity was observed as described previously ( Raymond & Fritsen, 2001). Briefly, 3 µl drops of supernatant were placed on a slide cover glass. A liquid nitrogen-cooled slide glued to a handle was pressed against the drops to form highly polycrystalline ice between the slide and cover glass. The samples were stored at -3°C in hexane and changes in recrystallization were monitored in the temperature bath described above over 21 hours. Photographs were taken through crossed polarizers.

### Sequencing

Green tissue was obtained and homogenized as described above. DNA was extracted with a NucleoSpin Plant II kit (Machery Nagel; catalog number 740770) according to the manufacturer’s instructions. To make IBP primers, the nucleotide sequences of IBPs from *L. perenne*, *D. antarctica* and *Triticum aestivum* (GenBank accession numbers EU680848, FJ663044 and KU204387, respectively) were aligned. Regions with high identities at the 5’ and 3’ ends were selected for primer sequences to obtain an amplicon that covered as much of the gene as possible. Primers for 18S ribosomal RNA were selected from conserved regions in the 18S sequences from *Dupontia fisheri*, *L. perenne* and *D. antarctica* (GenBank accession numbers KP794861, KJ598999 and MH628292, respectively) that flanked a region of high variability. At that time, we suspected the Utqiaġvik grass sample was *D. fisheri* and did not use the *L. mollis* 18S sequence. PCR was carried out with an Eppendorf Mastercycler Personal thermal cycler, with 3 minutes initial denaturation at 95°C, followed by 35 cycles of 30 seconds denaturation at 95°C, 30 seconds annealing at 59°C and 40 seconds extension at 72°C, followed by 3 minutes final extension at 72°C using Promega GoTaq polymerase (catalog number M300B). PCR products were electrophoresed on a 2% agarose gel, stained with ethidium bromide and observed and photographed with a UVP transilluminator. IBP and 18S bands of the expected sizes were obtained (see *Underlying data* ( Raymond, 2020c)), cleaned up with a Nucleospin gel and PCR clean-up kit (Machery Nagel, catalog number 740609) and sequenced in both directions at the UNLV Genomics Core with an Applied Biosystems 3130 sequencer. The primers that were used for sequencing are shown in Table 1.

**Table 1.** Primers used in this study.

Primer pair	Fwd	Rev	Size (bp)
IBP	5’-TGCCACCCCGATGACCTCCG-3’	5’-TTAACCTCCTGTCACGACTTTGTTGCTCCC-3’	798
18S	5’-GGAAGGATCATTGTCGTGACCCTGACC-3’	5’- CTGGGGTCGCGGTCGAAGCGTC-3’	623

IBP, ice-binding protein.

### Structure prediction

A 3D model of the IRI domain of the *Leymus* IBP was predicted with SWISS-MODEL ( Waterhouse *et al*., 2018) using the eight-coil structure of the *Lolium perenne* IRI domain (Protein Data Bank accession no. 3ULT) ( Middleton *et al*., 2012) as template. SWISS MODEL proposed three structures of the *Leymus* IRI domain based on the template. We selected the one with the highest sequence identity (76.5%), which also was the only one that had eight orderly coils like those in the *L. perenne* template. The free energy of this structure was then minimized (from -42.56 to -52.58 MJ mol ^-1^) with the YASARA Energy Minimization Server ( Krieger *et al*., 2009) and displayed with YASARA View. Stereoviews were obtained by rotating the molecule around its vertical axis by 3°. The average distance between coils in the IRI domain was measured with the YASARA distance function as the distance between the alpha carbons of Ser15 and Ser116 divided by seven.

## Results and discussion

### Species identification

The obtained 18S rRNA sequence (GenBank accession no. MT506010) most closely matched the sequence of *Leymus mollis* (GenBank accession no. EF581964) (98.3% identity). Among the Pooidae, *L. mollis* is a member of the Triticodae, which includes wheat ( *Triticum*) and barley ( *Hordeum*), while *Lolium* and *Deschampsia* are members of the Poodae. The grass was confirmed as *L. mollis* from photos of the remnant spikes and leaves by Matthew Carlson (University of Alaska Anchorage) and Carolyn Parker, University of Alaska Fairbanks (personal communications to T.S.).

### Ice-structuring and recrystallization inhibition activities

**Figure 1.**
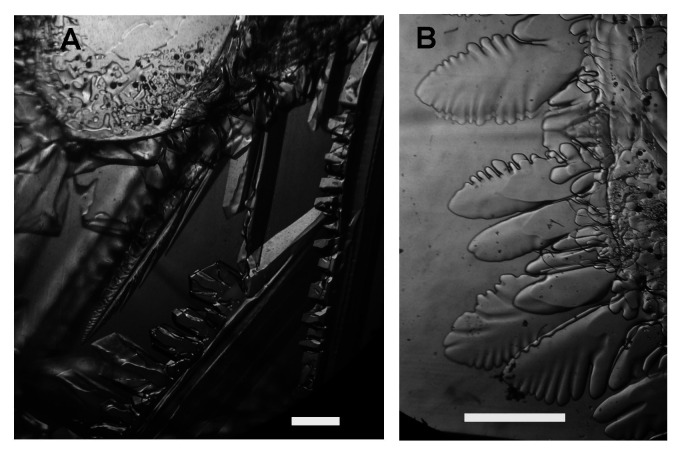
Ice-structuring abilities of grass extracts. ( **A**) *Leymus mollis* rhizome tissue. ( **B**) Lawn grass ( *Festuca* sp.) stem tissue (control). Ice seed crystals were placed in the extracts at slightly below the freezing point. Scale bars, 1 mm.

**Figure 2.**
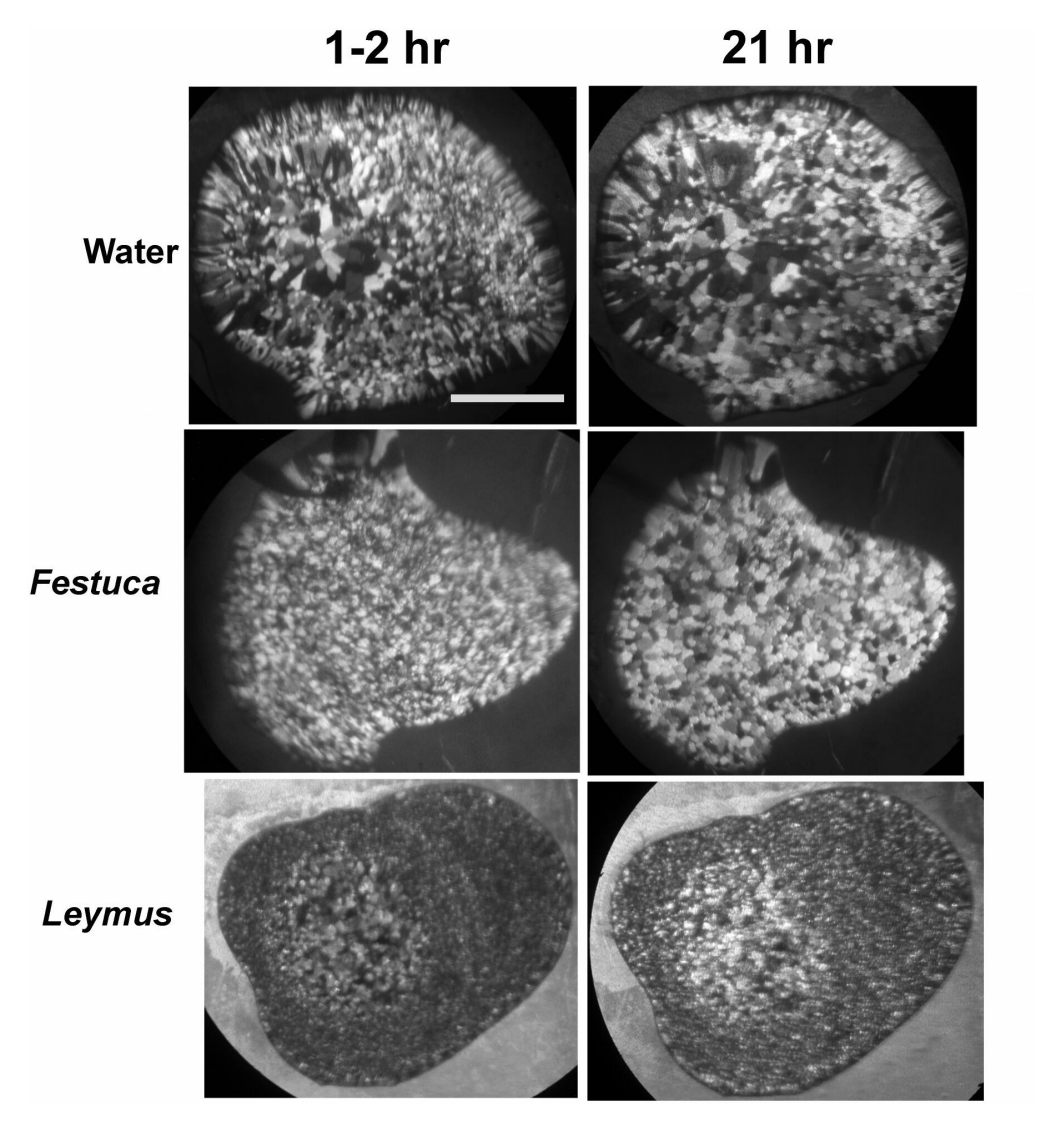
Comparison of ice recrystallization in 3 µl drops of water and extracts from lawn grass ( *Festuca*) and *L. mollis (Leymus)*. Scale bar, 1 mm.

An extract from green rhizome tissue strongly affected the growth of an ice seed crystal, causing it to develop sharp facets ( Figure 1A). The facets are an indication of the presence of ice-binding proteins. The facets themselves do not have any role in freezing tolerance, nor are they likely to form in frozen tissue; they are only a demonstration that molecules are binding to the ice surface, which is necessary for IRI. In constrast, ice grown in the presence of extract from lawn grass stem tissue (control) is smooth and rounded with no evidence of facet development ( Figure 1B). The *Leymus* extract also had strong IRI activity. Polycrystalline ice formed from water and the lawn grass extract recrystallized significantly after 21 hours at -3°C, while the supernatant of the *Leymus* extract showed no recrystallization ( Figure 2).

### Ice-binding protein

Primers based on the IBPs of other pooid grasses succeeded in amplifying a sequence that encoded the C-terminal part of the IBP gene of *L. mollis*, including the entire IRI domain. The sequence (GenBank accession no. MT506011) encoded 260 a.a., which corresponds to all but the first 25 a.a. of the *L. perenne* IBP sequence. The sequence included the stop codon and contained no introns. Although the sequence was close to the sequence of *L. perenne* (65% in the region of overlap), it most closely matched an IBP-like protein from *Triticum aestivum* (QBE94480) (86% identity, 91% similarity), in agreement with *L. mollis*’s classification as a member of the Triticodae. When only the IRI domains were compared, the *Leymus* IRI domain was also closer to the *Triticum* IRI domain than it was to the *Lolium* IRI domain. The ice-binding activities of *Tritium* spp. IBPs have not yet been reported.

**Figure 3.**
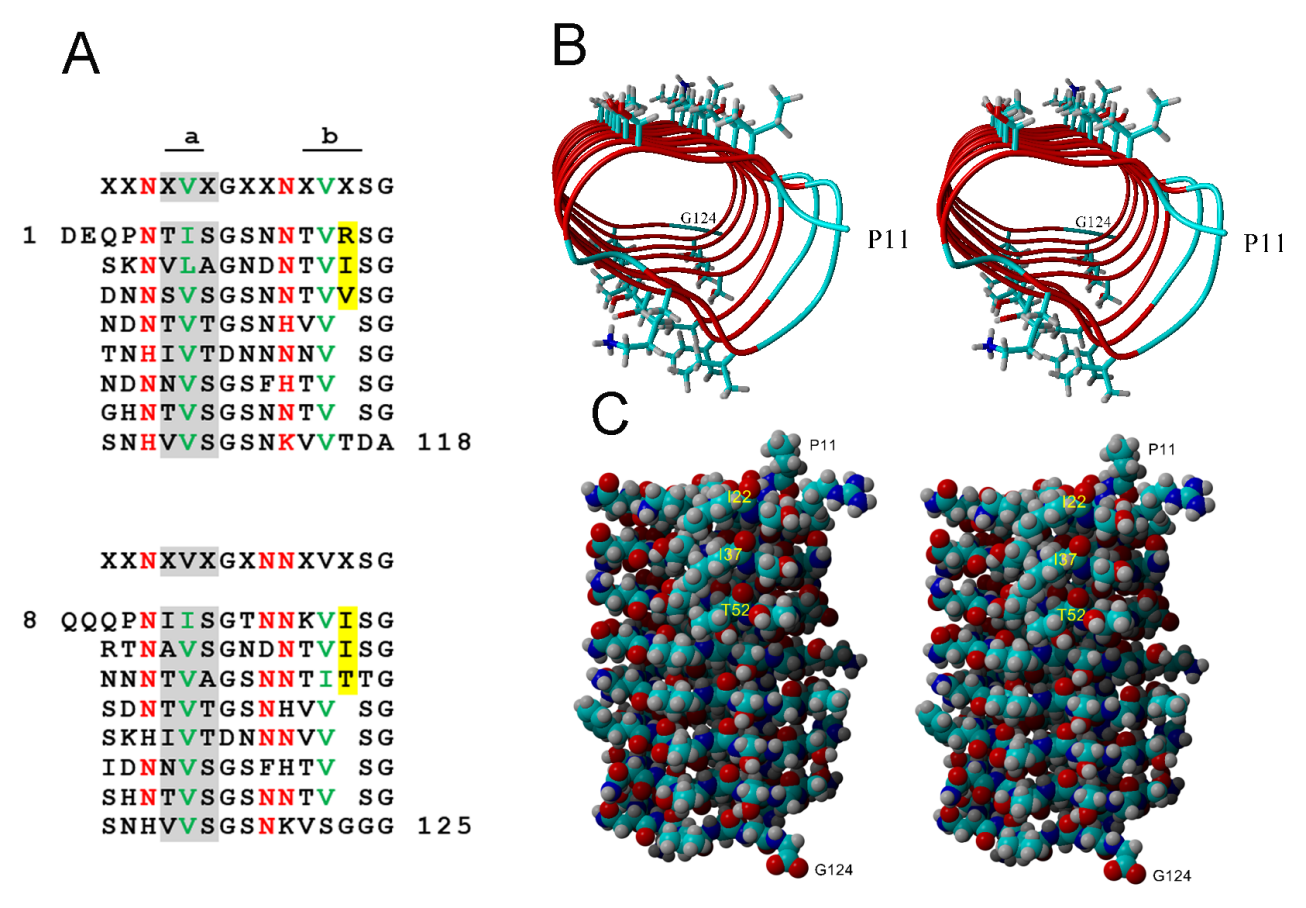
Structure of the IRI domain of *Leymus mollis* IBP. ( **A**) Comparison of the Leymus repeats (bottom) with those in the IRI domain of *Lolium perenne* (top). The *Lolium* data and the color scheme have been reproduced with permission from Middleton *et al*. (2012). Both domains have eight repeats. Consensus sequences are shown on top. Numbers indicate a.a. residue numbers. In the *Lolium* sequence, the gray background indicates the ice-binding site called the a side and the yellow background indicates a bulge on the b side. Similar features are found in the *Leymus* sequence. ( **B**, **C**) Stereoviews predicted by SWISS MODEL using the IRI domain of *L. perenne* as template. SWISS MODEL was able to model the *Leymus* structure from Pro11 to Gly124, which corresponds to Asp1 to Ala118 in *L. perenne*. ( **B**) View through the center of the coils, in which the ice-binding site (the a side) is on top. Amino acid side chains are shown only for the a and b sides. Color code of the ribbon: red, beta strand; cyan, coil. Color code of amino acid side chain: Cyan, C; blue, N, red, O; gray, H. ( **C**) View of the b side of a space-filling model. A bulge is created by two residues on each of the first three coils. The first of the two residues in each coil is labeled. Colors are the same as those for the side chains in **A**.

The IRI domain of *L. mollis*, like that of *L. perenne*, has eight repeats, each consisting of 14 or 15 residues ( Figure 3A). The consensus sequences of the two IBPs are virtually the same ( Figure 3A).

### Model of structure

The predicted structure of the *L. mollis* IBP IRI domain, obtained from using the *L. perenne* IBP IRI domain structure as template, resembles the *Lolium* IRI domain in several respects: it has eight repeats corresponding to eight coils in a beta-roll fold ( Figure 3B); a flat side (the a side) that is populated with two rows that are rich in Thr and Ser residues ( Figure 3B); an irregular side (the b side) that has a bulge in the first three coils ( Figure 3C); and an interior that is dominated by Asn/His ladders (red residues in Figure 3A). The a side has been identified as the ice-binding site of *L. perenne* IBP ( Middleton *et al.,* 2012). In *Lolium*, the average spacing between the alpha carbons of the row of amino acids on the ice-binding site is 4.5 Å, which is very similar to the spacing along the *a*-axis of ice (4.51 Å). In *Leymus*, the average spacing was calculated as 4.77 Å. The regular array of hydrophilic residues is thought to order water molecules that act as a glue between the IBP and the ice ( Middleton *et al.,* 2012).

In summary, we describe an ice-binding protein from the grass *Leymus mollis* with ice-structuring and ice recrystallization inhibition (IRI) activities. The collection site of the grass, a gravelly beach on the Chukchi Sea, is an extreme habitat subject to numerous environmental stresses. *L. mollis* appears to be better adapted to high latitudes than *L. perenne*, which does not grow well north of 60°N ( Helgadóttir *et al.,* 2018). It seems unlikely that the greater cold hardiness of *Leymus* can be attributed to its IBP as the IBPs of the two grasses are very similar. However, many proteins contribute to the cold hardiness of forage grasses, including transcription factors, cell membrane proteins, and proteins involved in cell signalling, cellular transport and photosynthesis ( Sandve *et al.,* 2011). It is thus likely that many of these factors are responsible for *L. mollis*’s survival in the high Arctic. One approach to identifying them would be a comparison of transcriptomes of the Arctic population with a California population that never experiences freezing conditions.

## Data availability

### Underlying data

*Leymus mollis* 18S ribosomal sequence on GenBank, Accession number MT506010

*Leymus mollis* IBP sequence on GenBank, Accession number MT506011

Figshare: Ice structuring by ice-binding protein of Leymus mollis DSCN5945.JPG. https://doi.org/10.6084/m9.figshare.12401966.v1 ( Raymond, 2020a)

Figshare: Leymus mollis ice recrsytallization inhibition. https://doi.org/10.6084/m9.figshare.12401954.v1 ( Raymond, 2020b)

Figshare: PCR amplification of IBP and 18S genes of Leymus mollis. https://doi.org/10.6084/m9.figshare.12401957.v1 ( Raymond, 2020c)

Data are available under the terms of the Creative Commons Attribution 4.0 International license (CC-BY 4.0).

### Extended data

*Figshare*: Table S1. Number of days with subzero air temperatures during the growing season at Utqiaġvik, AK. https://doi.org/10.6084/m9.figshare.12765983.v1 ( Raymond & Sformo, 2020)

This project contains the following extended data:

- Table S1.docx - Number of days with subzero air temperatures during the growing season at Utqiaġvik, AK. Data provided by the National Climatic Data Center.

Data are available under the terms of the Creative Commons Attribution 4.0 International license (CC-BY 4.0).
