# Supplementary material for: An ice-binding protein from an Arctic population of American dunegrass, Leymus mollis
Source: F1000Res. 2020 Aug 17;9:648. Originally published 2020 Jun 26. [Version 2] doi: 10.12688/f1000research.24328.2 (PMC7383708; doi:10.12688/f1000research.24328.2)
Supplement: Peer review history

# Peer review: Reviewer response for version 1

**DOI:** 10.5256/f1000research.26842.r65705

**Published:** 2020-07-27

**Authors:** Hans Ramløv

**Affiliations:** 1 Department of Science and Environment, Roskilde University, Roskilde, Denmark

- In the present paper Sformo and Raymond present data on an ice binding and recrystallisation inhibiting protein from the Alaskan grass Leymus mollis, which lives in areas where it is exposed to several kinds of stresses including low temperatures.
- The authors have observed ice structuring, recrystallisation inhibition activity in aqueous homogenates of the rhizome tissue. They have sequenced DNA from similar homogenates using primers based upon the conserved sequences from other IBP containing grasses. The obtained DNA sequences were translated into the amino acid sequences presumably involved in the ice recrystallisation inhibition and ice structuring. The amino acid sequence of the IRI domain of *L. mollis* was closely matched to the *Triticum aestivum* IRI domain.
- The 3D structure of the ice binding domain was predicted using the obtained sequence and the structure of the IRI domain in *Lolium perenne*. It appears that the tree dimensional structure of the ice binding domain of *L. mollis* IBP quite resembles that of *L. perenne*.
- The paper is an interesting comparative account of the ice related properties of a grass found at high latitudes - again indicating that ice structuring and recrystallisation inhibition is very important in plants adapted to cold climates.
- I miss an extension of the discussion of the comparative aspects of cold tolerance and adaptive aspects of the findings in the paper.
- Figure 1 could be explained a bit better as some readers may not understand the differences between the A and the B panel very well.
- Figure 2 is not very clear and also this figure would benefit from a bit more explanation in the text.

Is the work clearly and accurately presented and does it cite the current literature?

Yes

If applicable, is the statistical analysis and its interpretation appropriate?

Not applicable

Are all the source data underlying the results available to ensure full reproducibility?

Yes

Is the study design appropriate and is the work technically sound?

Yes

Are the conclusions drawn adequately supported by the results?

Yes

Are sufficient details of methods and analysis provided to allow replication by others?

Yes

Reviewer Expertise:

Cold tolerance of ectothermic animals, antifreeze proteins, cryptobiosis

I confirm that I have read this submission and believe that I have an appropriate level of expertise to confirm that it is of an acceptable scientific standard.

# Response

**Published:** 2020-07-30

**Authors:** James Raymond

**Affiliations:** University of Nevada Las Vegas, USA

•    I miss an extension of the discussion of the comparative aspects of cold tolerance and adaptive aspects of the findings in the paper.

*We revised the last paragraph to give some perspective to the cold tolerance of L. mollis and how it probably differs from that of L. perenne.*

•    Figure 1 could be explained a bit better as some readers may not understand the differences between the A and the B panel very well.

*We added some sentences to better explain the difference between Leymus and Festuca.*

•    Figure 2 is not very clear and also this figure would benefit from a bit more explanation in the text.

*We added new photos to show RI of Festuca. It more clearly shows that Leymus has much stronger RI than Festuca.*

# Peer review: Reviewer response for version 1

**DOI:** 10.5256/f1000research.26842.r65704

**Published:** 2020-07-23

**Authors:** Matthew Carlson

**Affiliations:** 1 Alaska Center for Conservation Science, University of Alaska Anchorage, Anchorage, AK, USA; 2 Biological Sciences, University of Alaska Anchorage, Anchorage, AK, USA

This brief report outlines a description of the sequence and structure of ice-binding protein in Leymus mollis from the Beaufort Coast in Arctic Alaska, and compares the findings to IBPs of other more temperate grass species.

 The paper seems pretty straightforward, however, I do not have the expertise to comment on the amino acid sequence, protein modeling, and comparative approach to other species. The paper was well-written, the figures were informative, and I believe it warrants indexing.

 While this is a brief note, I do think that additional interpretation of results and providing some brief explanations related to mechanisms of freeze tolerance in light of their findings would make the manuscript substantially more valuable. I was not able to gauge if the similarities and differences between Leymus and Lolium/Festuca are likely to have physiological/ecological bearing on their respective environmental contexts. (As an aside, since Leymus grows all down the Pacific Coast to San Diego it would be interesting to know if the IBP structure or concentrations/ability to upregulate are different along that substantial environmental gradient.).

 I provide additional comments and edits embedded as notes in a PDF - please see the file here.

 I appreciate the opportunity to read this paper and I sincerely hope that my comments are useful in generating a more robust paper.

Is the work clearly and accurately presented and does it cite the current literature?

Yes

If applicable, is the statistical analysis and its interpretation appropriate?

Not applicable

Are all the source data underlying the results available to ensure full reproducibility?

Yes

Is the study design appropriate and is the work technically sound?

Yes

Are the conclusions drawn adequately supported by the results?

Yes

Are sufficient details of methods and analysis provided to allow replication by others?

Yes

Reviewer Expertise:

Plant ecology and evolution - emphasis on Arctic and Boreal systems

I confirm that I have read this submission and believe that I have an appropriate level of expertise to confirm that it is of an acceptable scientific standard.

# Response

**Published:** 2020-07-27

**Authors:** James Raymond

**Affiliations:** University of Nevada Las Vegas, USA

Thank you for many good comments. Revisions have been made to address all of them.

# Response

**Published:** 2020-08-05

**Authors:** James Raymond

**Affiliations:** University of Nevada Las Vegas, USA

Thank you for many helpful comments. We revised the manuscript to address each of them. Our response to your comments can be viewed in the PDF file here.

# Peer review: Reviewer response for version 1

**DOI:** 10.5256/f1000research.26842.r65703

**Published:** 2020-07-20

**Authors:** Peter L. Davies

**Affiliations:** 1 Protein Function Discovery Group, Department of Biomedical and Molecular Sciences, Queen's University, Kingston, ON, Canada

Sformo and Raymond have prepared aqueous extracts of a hardy coastal grass ( *Leymus mollis*) collected in Alaska and have observed the presence of an ice-binding protein (IBP) based on ice crystal faceting and the inhibition of ice recrystallization. From another extract they prepared DNA and used primers based on conserved regions in other grass IBP transcripts to amplify the *L. mollis* gene, which fortunately lacks introns. From the sequence of this DNA they were able to provide the amino acid sequence of the IBP domain involved in ice recrystallization. They used this to model the three-dimensional structure of the domain, which looks very much like that of *Lollium perenne*. Another set of primers was used to amplify a diagnostic section of the 18S ribosomal RNA gene whose sequence identified the species.

 There is an element of comparative science here, and one would not need to see a repeat of this detailed analysis on other grasses/cereals. The presence of sequence homologs in transcriptomes as a way of seeing the distribution and variation of this IBP type should suffice from here on. Nevertheless, it is interesting to see this grass from a high latitude in Alaska (Beaufort Sea coast) extracted and analyzed. This report is also a good illustration of the use of molecular modeling to avoid solving a structure that would not differ much from the template. Indeed, the main features of *the Lolium perenne* IBP fold are retained in the *L. mollis* model, including the outward bulge of the beta-solenoid at one end on the non-ice-binding side.

 Suggestions for improvement:

- The Abstract should include the percent identity of the IRI domain between the two grass species. This type of information should also be provided in the Introduction where the sequences of the IBPs of *L. perenne* and *D. antarctica* are compared. It is not helpful to say their sequences “are very similar”. In the last paragraph of the Introduction the *L. mollis* IBP sequence is again said to be “very similar” to those of the other two grasses. Please provide % identity and % similarity.
- Figure 1A shows ice structuring. However, since panel B also shows ice crystals it is important to point out to the average reader that the distinction lies in the faceting seen in A where flat, angular surfaces are the result of IBP binding to ice. The ice in B is smoother and rounded.
- Figure 2 is a rather poor presentation of ice recrystallization. One must look closely at the ice control images to see the grains are larger after 21 h. The methods say the ice recrystallization was done in water. From our experience the inclusion of 150 mM NaCl makes a huge difference to the visibility of the ice grains. The other suggestion is to take the starting picture at zero time with the ice held colder than – 3 °C. We see substantial recrystallization happening in the first hour, even at -6 °C, and will typically hold the ice wafer at -10 °C until the starting picture is captured, and then raise the temperature for more rapid recrystallization. I recommend that this analysis be repeated and improved.
- In Methods, under ‘Structure prediction’, it is stated that three structures were proposed by SWISS MODEL operating with *L. perenne* IBP as a template. Then, the one with the ‘highest sequence identity’ was selected. Surely the sequence identity is the same for all models because they are based on the same template. We would typically compare models by root mean square deviation (RMSD) – how the atoms deviate from the model on average. Is this what is meant here?
- Although the sequence of the *L. mollis* open reading frame was incomplete, it would be useful to report the sequence upstream of the ice-binding domain. Presumably this is a series of leucine-rich repeats similar to those seen in other grass IBPs?
- In reference to ice crystal axes the a- and c- should be italicized.
- Replace the contraction "didn’t" with "did not".

Is the work clearly and accurately presented and does it cite the current literature?

Yes

If applicable, is the statistical analysis and its interpretation appropriate?

Not applicable

Are all the source data underlying the results available to ensure full reproducibility?

Yes

Is the study design appropriate and is the work technically sound?

Yes

Are the conclusions drawn adequately supported by the results?

Partly

Are sufficient details of methods and analysis provided to allow replication by others?

Yes

Reviewer Expertise:

Protein biochemistry, structural biology, ice-binding proteins

I confirm that I have read this submission and believe that I have an appropriate level of expertise to confirm that it is of an acceptable scientific standard.

# Response

**Published:** 2020-07-27

**Authors:** James Raymond

**Affiliations:** University of Nevada Las Vegas, USA

Percent identities added to Abstract. Identities were removed from Introduction to make a revision requested by reviewer 2.

Fig. 1A: The sentence was revised to point out that the ice is smooth and rounded with no evidence of facet development.

Fig. 2: The experiment requires much preparation and is difficult to repeat now that one of the authors is back in Alaska. However, we think there is a clear difference in recrystallization between the two samples when the figure is viewed at full scale.

Swiss-Model: Swiss Model tries different regions of the the sequence to model, e.g., residues 131-247 in one model and residues 144-228 in another. Each gives a slightly different model. We selected the one that had the highest a.a. sequence identity.

N-terminal region of IBP: For this, we would need a consensus sequence of the 5’ UTR in other grasses (for use as a forward primer) but we were unable to find such a sequence.

# Response

**Published:** 2020-07-28

**Authors:** James Raymond

**Affiliations:** University of Nevada Las Vegas, USA

Percent identity was added to Abstract and the Results. Identities were removed from Introduction to make a revision requested by reviewer 2.

Fig. 1A: The sentence was revised to point out that the ice is smooth and rounded with no evidence of facet development.

Fig. 2: We added a new control, Festuca (lawn grass) extract, which shows more clearly the greater RI activity of Leymus.

Swiss-Model: Swiss Model tries different regions of the sequence to model, e.g., residues 131-247 in one model and residues 144-228 in another. Each gives a slightly different model. We selected the one that had the highest a.a. sequence identity.

N-terminal region of IBP: For this, we would need a consensus sequence of the 5’ UTR in other grasses (for use as a forward primer) but we were unable to find such a sequence.
